# Supplementary material for: A Likelihood Approach for Real-Time Calibration of Stochastic Compartmental Epidemic Models
Source: PLoS Comput Biol. 2017 Jan 17;13(1):e1005257. doi: 10.1371/journal.pcbi.1005257 (PMC5240920; doi:10.1371/journal.pcbi.1005257)
Supplement: S1 File — (TAR.GZ) [file pcbi.1005257.s014.tar.gz › HSPH_Online-SI-Revision/Additions/PF09/n100-extreme_noisy/n100-extreme_noisy_PF2_table4.pdf]

| | | | | | | | | | | | | | | | | | | | | | | | | | |
|---|---|---|---|---|---|---|---|---|---|---|---|---|---|---|---|---|---|---|---|---|---|---|---|---|---|
| 1 | newly 3 weeks spec | 2278.3 | 4378.52 | {4275., 4495.} | 92.1833 | 0.283642 | newly 3 weeks cumul | 9885.83 | 15 346.7 | {15 205., 15 471.} | 55.239 | 0.190979 | newly 5 weeks spec | 1200.81 | 3017.28 | {2933., 3089.} | 151.269 | 0.399959 | newly 5 weeks cumul | 12 674.4 | 22 021.5 | {21 788., 22 170.} | 73.7474 | 0.239881 | newly 50 weeks cumul 18 298.5 33 988.9 {33 400., 34 335.} 85.7468 0.268808 |
| 2 | newly 3 weeks spec | 2435. | 3359.75 | {3263., 3442.} | 37.9774 | 0.139729 | newly 3 weeks cumul | 9225. | 12 221.5 | {12 113., 12 334.} | 32.4825 | 0.122145 | newly 5 weeks spec | 1337. | 2248.93 | {2176., 2296.} | 68.2072 | 0.225755 | newly 5 weeks cumul | 12 421. | 17 247. | {17 097., 17 379.} | 38.8532 | 0.142549 | newly 46 weeks cumul 16 331. 26 256.5 {26 082., 26 436.} 60.7772 0.206216 |
| 3 | newly 3 weeks spec | 1444. | 3044.26 | {2982., 3093.} | 110.821 | 0.323863 | newly 3 weeks cumul | 6755. | 12 083.8 | {12 001., 12 160.} | 78.8872 | 0.252572 | newly 5 weeks spec | 609. | 1776.6 | {1722., 1834.} | 191.724 | 0.464827 | newly 5 weeks cumul | 8307. | 16 198.3 | {16 117., 16 282.} | 94.9958 | 0.29002 | newly 51 weeks cumul 9551. 21 643.7 {21 512., 21 802.} 126.612 0.355276 |
| 4 | newly 3 weeks spec | 766. | 4225.73 | {4130., 4321.} | 451.662 | 0.741615 | newly 3 weeks cumul | 4576. | 16 588.9 | {16 463., 16 708.} | 262.52 | 0.559324 | newly 5 weeks spec | 254. | 2459.23 | {2395., 2513.} | 868.201 | 0.985891 | newly 5 weeks cumul | 5299. | 22 279.5 | {22 163., 22 393.} | 320.446 | 0.623707 | newly 55 weeks cumul 5634. 29 701.9 {29 587., 29 811.} 427.19 0.721965 |
| 5 | newly 3 weeks spec | 1978. | 4017.49 | {3942., 4092.} | 103.109 | 0.307681 | newly 3 weeks cumul | 9313. | 15 499.7 | {15 402., 15 623.} | 66.4306 | 0.221226 | newly 5 weeks spec | 894. | 2418.63 | {2366., 2482.} | 170.54 | 0.432141 | newly 5 weeks cumul | 11 542. | 21 053.8 | {20 940., 21 197.} | 82.4107 | 0.261046 | newly 52 weeks cumul 13 327. 28 734.6 {28 600., 28 862.} 115.612 0.333669 |
| 6 | newly 3 weeks spec | 2370. | 4260.54 | {4180., 4336.} | 79.7696 | 0.254669 | newly 3 weeks cumul | 9890. | 15 593.8 | {15 469., 15 712.} | 57.6728 | 0.197749 | newly 5 weeks spec | 1132. | 2711.12 | {2620., 2794.} | 139.498 | 0.379176 | newly 5 weeks cumul | 12 727. | 21 714.3 | {21 552., 21 878.} | 70.6161 | 0.232014 | newly 50 weeks cumul 15 600. 30 931.3 {30 725., 31 179.} 98.2772 0.297266 |
| 7 | newly 3 weeks spec | 1951. | 4889.03 | {4803., 4982.} | 150.591 | 0.398917 | newly 3 weeks cumul | 8547. | 16 530.9 | {16 384., 16 650.} | 93.4118 | 0.286475 | newly 5 weeks spec | 968. | 3596.5 | {3518., 3675.} | 271.539 | 0.569935 | newly 5 weeks cumul | 10 776. | 24 358.4 | {24 211., 24 492.} | 126.043 | 0.354186 | newly 48 weeks cumul 12 929. 40 487.8 {40 175., 40 793.} 213.155 0.495752 |
| 8 | newly 3 weeks spec | 2287. | 4178.14 | {4106., 4249.} | 82.6909 | 0.261677 | newly 3 weeks cumul | 9859. | 15 273.1 | {15 175., 15 366.} | 54.9151 | 0.190088 | newly 5 weeks spec | 1178. | 2697.06 | {2620., 2768.} | 128.952 | 0.359649 | newly 5 weeks cumul | 12 762. | 21 336. | {21 191., 21 461.} | 67.1837 | 0.223189 | newly 50 weeks cumul 15 717. 30 664.5 {30 430., 30 913.} 95.1037 0.290258 |
| 9 | newly 3 weeks spec | 2292. | 4801.24 | {4725., 4900.} | 109.478 | 0.321097 | newly 3 weeks cumul | 10 287. | 16 954.6 | {16 823., 17 053.} | 64.8159 | 0.216993 | newly 5 weeks spec | 1160. | 3230.58 | {3151., 3309.} | 178.498 | 0.444746 | newly 5 weeks cumul | 13 098. | 24 165.1 | {24 004., 24 311.} | 84.4944 | 0.265978 | newly 50 weeks cumul 15 554. 36 211.6 {35 956., 36 480.} 132.812 0.366999 |
| 10 | newly 3 weeks spec | 296. | 3831.7 | {3750., 3910.} | 1194.49 | 1.11204 | newly 3 weeks cumul | 1966. | 15 147.8 | {15 042., 15 263.} | 670.486 | 0.886758 | newly 5 weeks spec | 92. | 2213.73 | {2158., 2261.} | 2306.23 | 1.38126 | newly 5 weeks cumul | 2237. | 20 277.6 | {20 164., 20 368.} | 806.462 | 0.957346 | newly 54 weeks cumul 2337. 26 947.1 {26 817., 27 047.} 1053.06 1.06185 |
| 11 | newly 3 weeks spec | 2110. | 3207.12 | {3123., 3281.} | 51.9962 | 0.181766 | newly 3 weeks cumul | 9055. | 12 072.8 | {11 954., 12 195.} | 33.3276 | 0.124908 | newly 5 weeks spec | 1137. | 2002.35 | {1942., 2055.} | 76.1082 | 0.24566 | newly 5 weeks cumul | 11 827. | 16 628.5 | {16 473., 16 790.} | 40.5976 | 0.147966 | newly 48 weeks cumul 14 734. 23 501.3 {23 198., 23 861.} 59.5039 0.202743 |
| 12 | newly 3 weeks spec | 2018. | 5189.17 | {5107., 5270.} | 157.144 | 0.410141 | newly 3 weeks cumul | 9115. | 17 660.6 | {17 517., 17 773.} | 93.7527 | 0.287241 | newly 5 weeks spec | 959. | 3728.1 | {3643., 3816.} | 288.749 | 0.589604 | newly 5 weeks cumul | 11 517. | 25 824.2 | {25 673., 25 982.} | 124.227 | 0.350684 | newly 49 weeks cumul 13 641. 41 629.1 {41 387., 41 872.} 205.176 0.484546 |
| 13 | newly 3 weeks spec | 3118. | 4128.92 | {4036., 4226.} | 32.4221 | 0.121898 | newly 3 weeks cumul | 12 156. | 14 108.2 | {13 992., 14 213.} | 16.0598 | 0.0646735 | newly 5 weeks spec | 2008. | 3008.11 | {2938., 3080.} | 49.8063 | 0.175454 | newly 5 weeks cumul | 16 586. | 20 652.3 | {20 514., 20 774.} | 24.5163 | 0.0952214 | newly 47 weeks cumul 22 880. 34 217.2 {33 985., 34 436.} 49.5506 0.174783 |
| 14 | newly 3 weeks spec | 249. | 6596.66 | {6492., 6711.} | 2549.26 | 1.42309 | newly 3 weeks cumul | 1934. | 21 564.5 | {21 399., 21 729.} | 1015.02 | 1.04728 | newly 5 weeks spec | 37. | 4852.18 | {4746., 4962.} | 13 014. | 2.11768 | newly 5 weeks cumul | 2082. | 32 153.2 | {31 996., 32 310.} | 1444.34 | 1.18874 | newly 52 weeks cumul 2131. 51 460.6 {51 146., 51 754.} 2314.86 1.38289 |
| 15 | newly 3 weeks spec | 658. | 3822.27 | {3746., 3908.} | 480.892 | 0.764041 | newly 3 weeks cumul | 3705. | 14 837.8 | {14 688., 15 011.} | 300.479 | 0.602565 | newly 5 weeks spec | 197. | 2275.15 | {2211., 2337.} | 1054.9 | 1.06245 | newly 5 weeks cumul | 4246. | 20 066.8 | {19 907., 20 248.} | 372.605 | 0.67449 | newly 52 weeks cumul 4525. 27 203.9 {27 038., 27 350.} 501.192 0.779008 |
| 16 | newly 3 weeks spec | 902. | 4281.63 | {4191., 4360.} | 374.682 | 0.676354 | newly 3 weeks cumul | 5020. | 16 585.5 | {16 491., 16 676.} | 230.388 | 0.51902 | newly 5 weeks spec | 342. | 2549.67 | {2487., 2608.} | 645.518 | 0.872371 | newly 5 weeks cumul | 5877. | 22 441.4 | {22 332., 22 522.} | 281.851 | 0.581891 | newly 52 weeks cumul 6347. 30 335.3 {30 231., 30 452.} 377.946 0.679377 |
| 17 | newly 3 weeks spec | 2633. | 3775.4 | {3676., 3870.} | 43.3878 | 0.156437 | newly 3 weeks cumul | 10 424. | 13 605.6 | {13 492., 13 716.} | 30.5221 | 0.115674 | newly 5 weeks spec | 1461. | 2549. | {2480., 2610.} | 74.4695 | 0.241629 | newly 5 weeks cumul | 13 884. | 19 274.5 | {19 177., 19 374.} | 38.8249 | 0.142463 | newly 47 weeks cumul 18 108. 29 446.2 {29 206., 29 626.} 62.6144 0.211152 |
| 18 | newly 3 weeks spec | 1965. | 4597.33 | {4502., 4688.} | 133.961 | 0.369096 | newly 3 weeks cumul | 8760. | 14 824.6 | {14 697., 14 939.} | 69.2303 | 0.228469 | newly 5 weeks spec | 847. | 3636.72 | {3566., 3705.} | 329.365 | 0.632775 | newly 5 weeks cumul | 10 903. | 22 586.4 | {22 432., 22 739.} | 107.158 | 0.316294 | newly 48 weeks cumul 12 657. 41 428.2 {41 180., 41 717.} 227.315 0.51496 |
| 19 | newly 3 weeks spec | 2260. | 5264.05 | {5165., 5347.} | 132.923 | 0.367176 | newly 3 weeks cumul | 9867. | 17 924.8 | {17 784., 18 031.} | 81.6641 | 0.259262 | newly 5 weeks spec | 1026. | 3773.2 | {3685., 3854.} | 267.758 | 0.565499 | newly 5 weeks cumul | 12 362. | 26 191.1 | {26 026., 26 334.} | 111.867 | 0.32606 | newly 50 weeks cumul 14 680. 42 115.7 {41 912., 42 318.} 186.891 0.457714 |
| 20 | newly 3 weeks spec | 351. | 5781.77 | {5688., 5867.} | 1547.23 | 1.21672 | newly 3 weeks cumul | 2657. | 21 914.6 | {21 812., 22 012.} | 724.787 | 0.916338 | newly 5 weeks spec | 80. | 3479.04 | {3400., 3562.} | 4248.8 | 1.6383 | newly 5 weeks cumul | 2903. | 29 908.8 | {29 792., 30 017.} | 930.271 | 1.01295 | newly 55 weeks cumul 3010. 40 712.6 {40 582., 40 829.} 1252.58 1.13116 |
| 21 | newly 3 weeks spec | 1266. | 3777.43 | {3686., 3847.} | 198.375 | 0.474709 | newly 3 weeks cumul | 6452. | 14 698.7 | {14 585., 14 811.} | 127.816 | 0.357578 | newly 5 weeks spec | 504. | 2236.18 | {2174., 2298.} | 343.687 | 0.64699 | newly 5 weeks cumul | 7741. | 19 840.8 | {19 735., 19 937.} | 156.307 | 0.408757 | newly 52 weeks cumul 8578. 26 824.4 {26 705., 26 920.} 212.711 0.49514 |
| 22 | newly 3 weeks spec | 1097. | 3559.79 | {3480., 3635.} | 224.502 | 0.511158 | newly 3 weeks cumul | 5662. | 14 151.5 | {14 036., 14 255.} | 149.938 | 0.397824 | newly 5 weeks spec | 414. | 2046.95 | {1988., 2102.} | 394.432 | 0.693989 | newly 5 weeks cumul | 6768. | 18 908.1 | {18 780., 19 023.} | 179.375 | 0.446182 | newly 53 weeks cumul 7400. 25 036.4 {24 860., 25 253.} 238.33 0.529333 |
| 23 | newly 3 weeks spec | 2274. | 4201.72 | {4121., 4288.} | 84.7722 | 0.266582 | newly 3 weeks cumul | 9195. | 13 849.3 | {13 731., 13 969.} | 50.6181 | 0.177869 | newly 5 weeks spec | 1192. | 3257.98 | {3185., 3329.} | 173.32 | 0.436614 | newly 5 weeks cumul | 12 094. | 20 837.5 | {20 689., 20 980.} | 72.296 | 0.236269 | newly 46 weeks cumul 15 344. 37 697.8 {37 329., 37 988.} 145.685 0.390368 |
| 24 | newly 3 weeks spec | 2818. | 4338.83 | {4247., 4429.} | 53.9684 | 0.18737 | newly 3 weeks cumul | 11 319. | 14 154.4 | {14 012., 14 287.} | 25.0497 | 0.0970717 | newly 5 weeks spec | 1477. | 3388.88 | {3309., 3465.} | 129.443 | 0.360601 | newly 5 weeks cumul | 14 831. | 21 408.7 | {21 266., 21 562.} | 44.351 | 0.159414 | newly 46 weeks cumul 19 249. 39 246.5 {38 921., 39 582.} 103.889 0.309384 |
| 25 | newly 3 weeks spec | 1110. | 5464.62 | {5379., 5537.} | 392.308 | 0.692208 | newly 3 weeks cumul | 5663. | 17 704.9 | {17 559., 17 831.} | 212.642 | 0.49504 | newly 5 weeks spec | 432. | 4233.19 | {4136., 4318.} | 879.905 | 0.991126 | newly 5 weeks cumul | 6746. | 26 813.8 | {26 622., 26 981.} | 297.476 | 0.599305 | newly 49 weeks cumul 7332. 47 117.7 {46 830., 47 435.} 542.631 0.807957 |
| 26 | newly 3 weeks spec | 2919. | 3580.81 | {3488., 3660.} | 22.6725 | 0.0886769 | newly 3 weeks cumul | 11 387. | 13 076.4 | {12 959., 13 197.} | 14.8364 | 0.0600698 | newly 5 weeks spec | 1758. | 2359.36 | {2290., 2412.} | 34.2071 | 0.127658 | newly 5 weeks cumul | 15 461. | 18 351.6 | {18 236., 18 472.} | 18.6961 | 0.0744288 | newly 47 weeks cumul 21 236. 27 347.1 {27 164., 27 552.} 28.7771 0.10983 |
| 27 | newly 3 weeks spec | 2411. | 2971.23 | {2881., 3066.} | 23.2364 | 0.0905995 | newly 3 weeks cumul | 9503. | 11 254.3 | {11 122., 11 373.} | 18.4288 | 0.073432 | newly 5 weeks spec | 1418. | 1854.79 | {1782., 1946.} | 30.8032 | 0.116382 | newly 5 weeks cumul | 12 786. | 15 470.4 | {15 262., 15 662.} | 20.9952 | 0.0827342 | newly 48 weeks cumul 17 179. 21 754.2 {21 394., 22 167.} 26.6326 0.102486 |
| 28 | newly 3 weeks spec | 2066. | 2894.55 | {2829., 2964.} | 40.1041 | 0.146384 | newly 3 weeks cumul | 9289. | 11 581.4 | {11 413., 11 753.} | 24.6783 | 0.0957679 | newly 5 weeks spec | 1123. | 1669.12 | {1612., 1730.} | 48.6305 | 0.171946 | newly 5 weeks cumul | 12 043. | 15 457.4 | {15 287., 15 665.} | 28.3516 | 0.108382 | newly 52 weeks cumul 14 773. 20 477.7 {20 246., 20 726.} 38.6154 0.141794 |
| 29 | newly 3 weeks spec | 2511. | 4303.85 | {4203., 4381.} | 71.3998 | 0.233952 | newly 3 weeks cumul | 10 166. | 14 236.2 | {14 119., 14 340.} | 40.0373 | 0.146236 | newly 5 weeks spec | 1397. | 3304.06 | {3217., 3377.} | 136.511 | 0.373773 | newly 5 weeks cumul | 13 376. | 21 338. | {21 176., 21 462.} | 59.5241 | 0.202821 | newly 46 weeks cumul 17 318. 38 099.4 {37 813., 38 400.} 119.999 0.342412 |
| 30 | newly 3 weeks spec | 1594. | 4230.89 | {4147., 4319.} | 165.426 | 0.423892 | newly 3 weeks cumul | 7832. | 16 560.4 | {16 462., 16 655.} | 111.446 | 0.325194 | newly 5 weeks spec | 651. | 2469.25 | {2406., 2531.} | 279.301 | 0.578912 | newly 5 weeks cumul | 9505. | 22 259.5 | {22 173., 22 344.} | 134.187 | 0.36956 | newly 55 weeks cumul 10 576. 29 783.7 {29 657., 29 889.} 181.616 0.449656 |
| 31 | newly 3 weeks spec | 771. | 4960.99 | {4888., 5064.} | 543.449 | 0.808477 | newly 3 weeks cumul | 4574. | 19 233.2 | {19 121., 19 354.} | 320.489 | 0.623749 | newly 5 weeks spec | 189. | 2925.8 | {2855., 3000.} | 1448.04 | 1.18971 | newly 5 weeks cumul | 5139. | 25 984.7 | {25 855., 26 105.} | 405.637 | 0.703836 | newly 56 weeks cumul 5392. 34 870.4 {34 734., 34 993.} 546.706 0.810705 |
| 32 | newly 3 weeks spec | 858. | 3426.85 | {3341., 3512.} | 299.4 | 0.601336 | newly 3 weeks cumul | 4649. | 13 603.7 | {13 476., 13 714.} | 192.615 | 0.466286 | newly 5 weeks spec | 288. | 1976.43 | {1907., 2042.} | 586.26 | 0.836351 | newly 5 weeks cumul | 5438. | 18 208.9 | {18 111., 18 356.} | 234.846 | 0.524839 | newly 53 weeks cumul 5832. 24 207.9 {24 083., 24 403.} 315.088 0.618133 |
| 33 | newly 3 weeks spec | 3068. | 3453.12 | {3351., 3568.} | 12.5528 | 0.0512265 | newly 3 weeks cumul | 12 287. | 13 524.5 | {13 262., 13 836.} | 10.0719 | 0.0416147 | newly 5 weeks spec | 1670. | 2036.65 | {1942., 2117.} | 21.9551 | 0.0859745 | newly 5 weeks cumul | 16 122. | 18 231.7 | {17 870., 18 665.} | 13.0855 | 0.053343 | newly 51 weeks cumul 20 713. 24 632.3 {24 077., 25 290.} 18.9217 0.0751786 |
| 34 | newly 3 weeks spec | 2614. | 3618.2 | {3545., 3687.} | 38.4162 | 0.14113 | newly 3 weeks cumul | 10 179. | 12 800.7 | {12 668., 12 904.} | 25.7556 | 0.0995131 | newly 5 weeks spec | 1528. | 2508.86 | {2439., 2575.} | 64.1924 | 0.215247 | newly 5 weeks cumul | 13 712. | 18 341.6 | {18 183., 18 520.} | 33.7628 | 0.126324 | newly 46 weeks cumul 18 212. 28 919. {28 545., 29 243.} 58.7906 0.200806 |
| 35 | newly 3 weeks spec | 2810. | 4670.74 | {4574., 4769.} | 66.2185 | 0.220629 | newly 3 weeks cumul | 11 877. | 17 011.6 | {16 903., 17 116.} | 43.2317 | 0.156034 | newly 5 weeks spec | 1428. | 2971.07 | {2906., 3037.} | 108.058 | 0.31812 | newly 5 weeks cumul | 15 254. | 23 741.3 | {23 627., 23 877.} | 55.6396 | 0.192116 | newly 51 weeks cumul 18 586. 33 659. {33 429., 33 854.} 81.0985 0.25791 |
| 36 | newly 3 weeks spec | 2377. | 3005.19 | {2911., 3092.} | 26.4279 | 0.101739 | newly 3 weeks cumul | 9678. | 11 573.2 | {11 375., 11 748.} | 19.5822 | 0.0776333 | newly 5 weeks spec | 1295. | 1810.05 | {1742., 1880.} | 39.7722 | 0.145227 | newly 5 weeks cumul | 12 751. | 15 728.1 | {15 469., 15 972.} | 23.348 | 0.0910949 | newly 49 weeks cumul 16 300. 21 451.4 {21 021., 21 927.} 31.6038 0.119212 |
| 37 | newly 3 weeks spec | 918. | 3879.93 | {3806., 3956.} | 322.65 | 0.625931 | newly 3 weeks cumul | 5030. | 15 285.8 | {15 180., 15 376.} | 203.893 | 0.482715 | newly 5 weeks spec | 306. | 2260.28 | {2203., 2319.} | 638.654 | 0.868335 | newly 5 weeks cumul | 5864. | 20 505.1 | {20 400., 20 593.} | 249.678 | 0.543665 | newly 54 weeks cumul 6382. 27 373.2 {27 270., 27 469.} 328.913 0.632367 |
| 38 | newly 3 weeks spec | 286. | 5232.3 | {5140., 5311.} | 1729.48 | 1.26229 | newly 3 weeks cumul | 2079. | 20 043.8 | {19 921., 20 179.} | 864.108 | 0.984121 | newly 5 weeks spec | 76. | 3121.29 | {3032., 3192.} | 4006.96 | 1.61343 | newly 5 weeks cumul | 2297. | 27 238.2 | {27 105., 27 355.} | 1085.82 | 1.07401 | newly 54 weeks cumul 2347. 36 872.8 {36 754., 36 993.} 1471.06 1.19619 |
| 39 | newly 3 weeks spec | 1569. | 4066.75 | {3976., 4147.} | 159.194 | 0.413567 | newly 3 weeks cumul | 6961. | 14 825.9 | {14 694., 14 938.} | 112.985 | 0.32834 | newly 5 weeks spec | 719. | 2671.52 | {2598., 2748.} | 271.561 | 0.569921 | newly 5 weeks cumul | 8738. | 20 809.8 | {20 675., 20 935.} | 138.153 | 0.376851 | newly 48 weeks cumul 10 021. 30 852.5 {30 620., 31 083.} 207.878 0.488371 |
| 40 | newly 3 weeks spec | 2069. | 3025.82 | {2926., 3121.} | 46.2455 | 0.164934 | newly 3 weeks cumul | 8421. | 11 869. | {11 646., 12 119.} | 40.945 | 0.149002 | newly 5 weeks spec | 1045. | 1782.37 | {1714., 1869.} | 70.5617 | 0.23161 | newly 5 weeks cumul | 10 908. | 15 992.3 | {15 627., 16 333.} | 46.6107 | 0.166105 | newly 49 weeks cumul 13 560. 21 482. {20 941., 22 039.} 58.4218 0.199724 |
| 41 | newly 3 weeks spec | 1243. | 2981.2 | {2761., 3192.} | 139.839 | 0.378866 | newly 3 weeks cumul | 5945. | 11 741.6 | {11 101., 12 438.} | 97.5045 | 0.294903 | newly 5 weeks spec | 608. | 1711.65 | {1542., 1897.} | 181.521 | 0.447772 | newly 5 weeks cumul | 7464. | 15 723. | {14 822., 16 770.} | 110.651 | 0.322748 | newly 50 weeks cumul 8541. 20 687.3 {19 153., 22 411.} 142.212 0.382905 |
| 42 | newly 3 weeks spec | 457. | 3855.55 | {3776., 3931.} | 743.665 | 0.926118 | newly 3 weeks cumul | 2671. | 15 212.2 | {15 100., 15 320.} | 469.533 | 0.755512 | newly 5 weeks spec | 82. | 2238.52 | {2176., 2311.} | 2629.9 | 1.43603 | newly 5 weeks cumul | 2987. | 20 393.6 | {20 278., 20 496.} | 582.745 | 0.834255 | newly 54 weeks cumul 3092. 27 157.2 {27 049., 27 273.} 778.306 0.943644 |
| 43 | newly 3 weeks spec | 2726. | 4707.8 | {4614., 4780.} | 72.6999 | 0.237246 | newly 3 weeks cumul | 11 008. | 14 633.6 | {14 509., 14 755.} | 32.936 | 0.123634 | newly 5 weeks spec | 1377. | 3990.66 | {3902., 4071.} | 189.808 | 0.462047 | newly 5 weeks cumul | 14 359. | 23 012.1 | {22 836., 23 165.} | 60.2626 | 0.204824 | newly 47 weeks cumul 18 000. 46 702.3 {46 377., 47 022.} 159.457 0.41406 |
| 44 | newly 3 weeks spec | 1026. | 3386.28 | {3320., 3446.} | 230.047 | 0.518523 | newly 3 weeks cumul | 5335. | 13 433.4 | {13 345., 13 526.} | 151.797 | 0.401043 | newly 5 weeks spec | 393. | 1953.75 | {1889., 2008.} | 397.137 | 0.696339 | newly 5 weeks cumul | 6350. | 17 975. | {17 903., 18 055.} | 183.071 | 0.451893 | newly 53 weeks cumul 7041. 23 923.7 {23 812., 24 028.} 239.778 0.531192 |
| 45 | newly 3 weeks spec | 374. | 4232.05 | {4152., 4304.} | 1031.56 | 1.05364 | newly 3 weeks cumul | 2546. | 16 415.3 | {16 301., 16 532.} | 544.747 | 0.809383 | newly 5 weeks spec | 135. | 2514.25 | {2453., 2584.} | 1762.41 | 1.26999 | newly 5 weeks cumul | 2913. | 22 208.6 | {22 103., 22 291.} | 662.396 | 0.882178 | newly 52 weeks cumul 3058. 30 006.7 {29 884., 30 133.} 881.253 0.991779 |
| 46 | newly 3 weeks spec | 1322. | 4503.64 | {4427., 4588.} | 240.669 | 0.532296 | newly 3 weeks cumul | 7028. | 17 203.8 | {17 080., 17 312.} | 144.79 | 0.388788 | newly 5 weeks spec | 520. | 2713.34 | {2647., 2766.} | 421.796 | 0.717418 | newly 5 weeks cumul | 8416. | 23 432.1 | {23 296., 23 541.} | 178.423 | 0.444702 | newly 52 weeks cumul 9218. 32 117.8 {31 988., 32 256.} 248.425 0.542107 |
| 47 | newly 3 weeks spec | 877. | 3819.98 | {3732., 3907.} | 335.574 | 0.638998 | newly 3 weeks cumul | 5116. | 15 087.6 | {14 973., 15 204.} | 194.91 | 0.469682 | newly 5 weeks spec | 280. | 2219.05 | {2164., 2279.} | 692.518 | 0.89892 | newly 5 weeks cumul | 5861. | 20 221. | {20 102., 20 334.} | 245.009 | 0.537824 | newly 54 weeks cumul 6385. 26 917.9 {26 757., 27 057.} 321.58 0.624876 |
| 48 | newly 3 weeks spec | 1336. | 4610.97 | {4527., 4697.} | 245.132 | 0.537942 | newly 3 weeks cumul | 7014. | 18 020. | {17 897., 18 127.} | 156.915 | 0.409783 | newly 5 weeks spec | 498. | 2701.71 | {2634., 2757.} | 442.512 | 0.734345 | newly 5 weeks cumul | 8264. | 24 277.1 | {24 156., 24 395.} | 193.769 | 0.468003 | newly 54 weeks cumul 8948. 32 424.7 {32 320., 32 539.} 262.368 0.559148 |
| 49 | newly 3 weeks spec | 215. | 5058.64 | {4978., 5146.} | 2252.86 | 1.37156 | newly 3 weeks cumul | 1583. | 19 592.2 | {19 456., 19 730.} | 1137.66 | 1.0926 | newly 5 weeks spec | 43. | 2993.8 | {2918., 3061.} | 6862.33 | 1.84269 | newly 5 weeks cumul | 1735. | 26 493.3 | {26 345., 26 610.} | 1426.99 | 1.18383 | newly 56 weeks cumul 1768. 35 619.6 {35 522., 35 733.} 1914.68 1.3042 |
| 50 | newly 3 weeks spec | 2185. | 3940.42 | {3862., 4034.} | 80.3396 | 0.256036 | newly 3 weeks cumul | 9176. | 13 926.1 | {13 807., 14 037.} | 51.7668 | 0.181169 | newly 5 weeks spec | 1236. | 2722.9 | {2649., 2792.} | 120.299 | 0.342929 | newly 5 weeks cumul | 12 158. | 19 941.7 | {19 810., 20 059.} | 64.0212 | 0.214895 | newly 47 weeks cumul 15 907. 31 350.8 {31 158., 31 562.} 97.0882 0.294655 |
